# Diabetes Prevention Program Sites Compared With Diabetes Prevalence and Ratio of Primary Care Physicians in Texas

**DOI:** 10.5888/pcd16.190175

**Published:** 2019-12-26

**Authors:** Emily Peterson Johnson, Melissa Dunn, Maria Cooper, Nimisha Bhakta

**Affiliations:** 1 Texas Department of State Health Services, Austin, Texas

**Figure:**
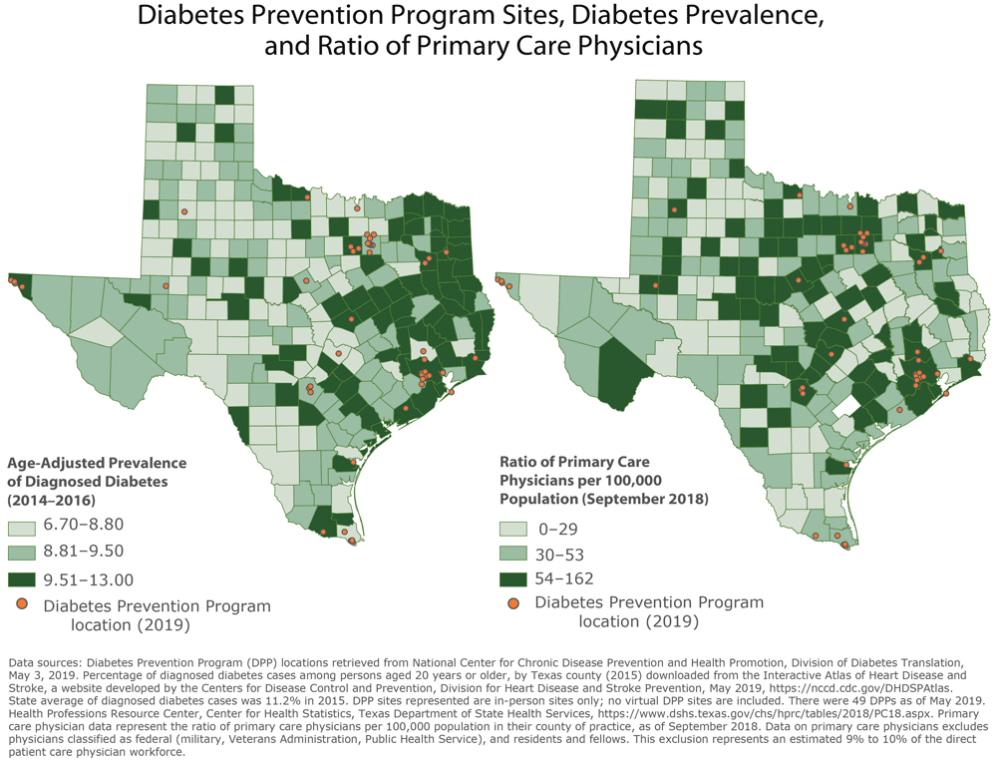
These maps show the prevalence of diabetes and the ratio of primary care physicians in each county in Texas. Additionally, the maps display the locations of the 49 Diabetes Prevention Program (DPP) sites throughout Texas, as of May 3, 2019. These maps can be used by the Texas Department of State Health Services, health care organizations, or health care providers to identify priority locations for new DPP sites.

## Background

The prevalence of diabetes has gradually increased over the past 10 years, both in Texas and nationally (1). In 2017, an estimated 2,323,220 people in Texas had diabetes, which represented 11.4% of the adult population (2). Additionally, approximately 23.8% of people who had diabetes were not aware of it (3).

The total direct medical expenses for diagnosed diabetes in Texas in 2012 was $18.9 billion, while indirect costs for things such as increased absenteeism, reduced productivity, or inability to work as a result of diabetes was an additional $6.7 billion (4). Texas is among the 10 states collectively responsible for over 50% of the national cost of diabetes (3).

In 2018, the Centers for Disease Control and Prevention (CDC) awarded the Texas Department of State Health Services (DSHS) the DP18–1815 cooperative agreement to address diabetes prevention and management. With this award, DSHS is partnering with organizations across the state to implement and increase access to CDC-recognized Diabetes Prevention Programs (DPPs), which provide evidence-based education for type 2 diabetes prevention. The DPP has been shown to significantly prevent or delay diabetes, even for many years after completion (5).

## Data Sources and Map Logistics

Though prevalence of prediabetes is an important indicator for planning DPP sites, the Texas Behavioral Risk Factor Survelliance System (BRFSS) does not have direct estimates of prediabetes prevalence for all Texas counties because of insufficient sample size. Additionally, these estimates have not been published as predicted estimates from CDC’s BRFSS small area estimation effort. As such, the prevalence of diagnosed diabetes was used to create this map. County-level estimates of diagnosed diabetes among adults aged 20 years or older were determined by using data from the CDC’s BRFSS (6) and the US Census Bureau’s Population Estimates Program (7). Diagnosed diabetes was defined as a “yes” response to the BRFSS survey question, “Has a doctor ever told you that you have diabetes?” Women who indicated they had had diabetes only in pregnancy were excluded.

The data in these maps represent year-specific and county-level estimates of diabetes prevalence in Texas in 2015. Three years of data (2014–2016) were used to improve the precision of county-level estimates. County-level estimates were calculated by using indirect model-dependent estimates, which used Bayesian multilevel modeling techniques. Age adjustments were calculated based on the US population in 2000 by using age groups 20 to 44 years, 45 to 64 years, and 65 years or older. These analyses were performed by CDC, and data are publicly available. DSHS retrieved the data on May 23, 2019, from the Interactive Atlas of Heart Disease and Stroke, a website developed by the CDC Division for Heart Disease and Stroke Prevention (8).

Data for the locations of DPPs were retrieved from the National Center for Chronic Disease Prevention and Health Promotion, Division of Diabetes Translation, on May 3, 2019 (9). Virtual DPP sites were not included on the map; only in-person or combination sites are displayed. These sites were downloaded as a .csv file, which included zip code and latitude and longitude for each location. A Python code was used to clean the spreadsheet characters that were corrupted in the download. The table was then geocoded and visualized using ArcGISPro (Esri).

Data for the ratio of primary care physicians (PCPs) were downloaded from the DSHS Health Professions Resource Center (10). The data represent the ratio of PCPs per 100,000 population in their county of practice as of September 2018. Physicians were not included in the count if they were classified as federal, such as those in the military, Veterans Administration, Public Health Service, or residents and fellows. This exclusion represents approximately 9% to 10% of the physician workforce.

## Highlights

These maps allow readers to identify the geographic spread of DPPs in comparison to diagnosed diabetes prevalence and the ratio of primary care physicians. A key finding of these maps is that East Texas has a higher prevalence of diagnosed diabetes compared with the overall state. Many of the counties in East Texas that have a high prevalence are rural or semirural. Additionally, several clusters of counties in North Texas and along the US-Mexico border have a high prevalence of diagnosed diabetes.

Although there is a concentration of DPP sites throughout East Texas, many of the counties with the highest rates of diagnosed diabetes do not have DPP sites at all, nor are there sites in nearby counties. Additionally, many counties in East Texas have low ratios of primary care physicians.

The main limitation of these maps is that they used county-level estimates of diagnosed diabetes from 2015. Although it is likely that the prevalence has changed in the last 4 years, these data represent the most recent age-adjusted, county-level estimates for all 254 counties in Texas. These maps, however, can still be used to understand the overall prevalence and geographic distribution of diabetes and available services to address diabetes in Texas.

## Action

These maps can serve as a powerful tool for planning diabetes prevention and education initiatives in Texas. DSHS and its partners will use these maps to pinpoint priority counties in which to establish new DPP sites, with particular attention to counties that also have low ratios of PCPs. These maps will also inform the work DSHS is doing with regional partners to expand coverage of the DPP through employer-provided health insurance. Once locations for new DPP sites have been identified, the same areas can be targeted for expanding the coverage of DPP by employer-based insurance, to make DPP classes more financially accessible.

These maps also have application for health care providers and health care organizations working throughout Texas. These stakeholders can use these maps to understand the health care needs of the populations they serve, inform program planning or expansion of services, and develop regional partnerships, particularly in areas with high prevalence of diagnosed diabetes and lower access to both DPPs and PCPs.
